# P03-07 Do Short-term Exercise Interventions Improve Traditional Cardiometabolic Disease Risk Factors in Children?

**DOI:** 10.1093/eurpub/ckac095.043

**Published:** 2022-08-29

**Authors:** Anneke van Biljon

**Affiliations:** Human Movement Science, University of Zululand, KwaDlangezwa, South Africa

**Keywords:** Exercise intensity, high-intensity interval training, moderate-intensity continuous training

## Abstract

**Background:**

This study aimed to explore the impact of short-term exercise of varying intensity on traditional cardiometabolic disease (CMD) risk factors.

**Methods:**

One hundred-and-nine children (11.07 ± 0.81 y) were conveniently assigned to 5-weeks of either: moderate intensity continuous training (MICT; n = 29) set at 65% - 70% of maximum heart rate (MHR); high intensity interval training (HIIT; n = 29; > 80% MHR); combined training (HIIT + MICT; n = 27); or no training (CT; n = 24). A two-way analysis of variance (group x time) was used to evaluate the effects of training on all traditional CMD risk parameters. Effect sizes (ES) were calculated to assess the magnitude of difference.

**Results:**

MICT, HIIT and HIIT + MICT significantly improved resting heart rate (ES = -0.39; ES = -1.05; ES = -1.05; p > 0.0001), fasting glucose (ES = -0.63; ES = -0.90; ES = -0.13; p = 0.0004), peak oxygen consumption (ES = 0.53; ES = 0.88; ES = 0.46; p > 0.0001) and c-reactive protein (ES = -0.18; ES = -1.04; ES = -0.54; p = 0.0016), respectively. The HIIT + MICT group significantly reducedwaist circumference(-5.37%; p > 0.0001) and waist-to-hip ratio (-2.47%; p > 0.0002) compared with the MICT (6.99%; 6.33%) and HIIT (-0.50%; -1.27%) groups, respectively.

**Conclusion:**

The findings from this study indicate that short-term HIIT and MICT interventions are both effective for improving cardiometabolic health in children. HIIT + MICT may provide superior reductions in central obesity indicators.

